# The clinical features and genomic epidemiology of carbapenem-resistant *Acinetobacter baumannii* infections at a tertiary hospital in Vietnam

**DOI:** 10.1016/j.jgar.2023.04.007

**Published:** 2023-06

**Authors:** Duong Thi Hong Diep, Huynh Minh Tuan, Kha My Ngoc, Chau Vinh, Tran Thi Ngoc Dung, Voong Vinh Phat, Quynh Nguyen, Dong Thi Hoai Tam, Lam Vinh Nien, Bui Thi Hanh Duyen, Cao Thi Phung, Nguyen Hoang Bac, Tran Diep Tuan, Guy Thwaites, Maia A. Rabaa, Duy Thanh Pham

**Affiliations:** a University of Medicine and Pharmacy, Ho Chi Minh City, Vietnam; b University Medical Center, Ho Chi Minh City, Vietnam; c Oxford University Clinical Research Unit, Ho Chi Minh City, Vietnam; d Centre for Tropical Medicine and Global Health, Nuffield Department of Clinical Medicine, Oxford University, United Kingdom

**Keywords:** *Acinetobacter baumannii*, Carbapenem resistance, Whole genome sequencing, Hospital-acquired infections, Nosocomial infections

## Abstract

**Objectives:**

To characterise the clinical features of *Acinetobacter baumannii* infections and investigate the phylogenetic structure and transmission dynamics of *A. baumannii* in Vietnam.

**Methods:**

Between 2019 and 2020, a surveillance of *A. baumannii* (AB) infections was conducted at a tertiary hospital in Ho Chi Minh City, Vietnam. Risk factors for in-hospital mortality were analysed using logistic regressions. Whole-genome sequence data were used to characterise genomic species, sequence types (STs), antimicrobial resistance genes, surface antigens, and phylogenetic relatedness of AB isolates.

**Results:**

Eighty-four patients with AB infections were enrolled in the study, 96% of whom were hospital-acquired. Half of the AB isolates were identified from ICU-admitted patients, while the remaining isolates were from non-ICU patients. The overall in-hospital mortality was 56%, with associated risk factors including advanced age, ICU stay, exposure to mechanical ventilation/central venous catheterization, pneumonia as source of AB infection, prior use of linezolid/aminoglycosides, and AB treatment with colistin-based therapy. Nearly 91% of isolates were carbapenem-resistant; 92% were multidrug-resistant; and 6% were colistin-resistant. ST2, ST571, and ST16 were the three dominant carbapenem-resistant *A. baumannii* (CRAB) genotypes, exhibiting distinct AMR gene profiles. Phylogenetic analysis of CRAB ST2 isolates together with previously published ST2 collection provided evidence of intra- and inter-hospital transmission of this clone.

**Conclusions:**

Our study highlights a high prevalence of carbapenem resistance and multidrug resistance in *A. baumannii* and elucidates the spread of CRAB within and between hospitals. Strengthening infection control measures and routine genomic surveillance are crucial to reducing the spread of CRAB and detecting novel pan-drug-resistant variants in a timely fashion.

## Highlights

• Rate of carbapenem resistance in *Acinetobacter baumannii* is extremely high in Vietnam.
• *Acinetobacter baumannii* nosocomial infections are not limited to intensive care units.
• Genomic survey detects intra- and inter-hospital spread of *Acinetobacter baumannii.*

## 1 Introduction

*Acinetobacter baumannii*-*calcoaceticus* complex is a group of aerobic, non-fermentative, Gram-negative coccobacilli, comprising of four different species: *A. baumannii, A. pittii, A. nosocomialis*, and *A. calcoaceticus*. Since the 1960s–1970s, *Acinetobacter* have emerged as one of the leading causes of nosocomial infections, causing bloodstream infections (BSIs), hospital-acquired pneumonia (HAP), and ventilator-associated pneumonia (VAP), especially in ICUs worldwide [1,2]. Major predisposing factors include prolonged hospital stay, invasive procedures, advanced age, immunosuppression, and exposure to broad-spectrum antimicrobials, particularly in ICU patients [3], [4], [5], [6]. *Acinetobacter* are intrinsically resistant to desiccants, disinfectants, and key antimicrobials, which contributes significantly to their long-term persistence and transmission in healthcare environments.

Within the *Acinetobacter baumannii*-*calcoaceticus* complex, *A. baumannii* has been identified as a priority pathogen by the WHO for research and development of new antimicrobials, underlining its significant threat to global public health [6]. Carbapenem-resistant *A. baumannii* (CRAB) are especially challenging for clinical management, given the lack of effective treatment drugs [7]. Furthermore, multiple lineages of CRAB have emerged independently followed by international spread, often with an XDR phenotype (defined as resistance to all antimicrobials other than polymyxins and tigecycline). Previous studies have shown that antimicrobial resistance (AMR) is the most important determinant of clinical outcome in *A. baumannii* infections, and ineffective early therapy is associated with increased mortality [6]. XDR *A. baumannii* infections may be treated with tigecycline or colistin; however, these drugs are less effective, with high toxicity and increasing resistance reported [8].

Carbapenem resistance in *A. baumannii* is mediated by the interaction of multiple mechanisms; specifically, the overexpression of plasmid- and/or chromosomal-mediated *bla*_OXA_ genes encoding oxacillinases (*bla*_OXA-23_, *bla*_OXA-24/40_, *bla*_OXA-51_, *bla*_OXA-58_, and *bla*_OXA-143_); the presence of metallo-carbapenemases (*bla*_IMP_, *bla*_VIM_, *bla*_SIM_, and *bla*_NDM_), and downregulation of porins constituting channels for the influx of carbapenems [9]. There are two major globally disseminated clones of *A. baumannii*, known as GC1 and GC2, which emerged and became resistant to older drugs in the 1970s, followed by further resistance to newer drugs (fluoroquinolones, cephalosporins, and carbapenems) in the 1980s [10,11]. Currently, GC2 has successfully disseminated throughout Asia [12], Europe [13], Australia [14], and the United States [15] and appears to have become predominant in Asia [16,17]. The *bla*_OXA-23_–carrying CRAB that have been reported worldwide belong primarily to GC2.

A recent systematic review has underlined high incidence and increased mortality in hospital-acquired infections (HAIs) caused by CRAB in Southeast Asia [18]. In Vietnam, *A. baumannii* represents a leading cause of HAIs, resulting in significant health burden. Data from studies across Vietnam have demonstrated a dramatic increase of CRAB since 2009, predominantly associated with VAP and BSIs [19], [20], [21], [22], [23], [24], [25]. The proportion of CRAB was reported to range from 55% to 90% [24], [25], [26] with mortality rates up to 52% reported in VAP patients of a tertiary hospital [20]. XDR was also common; for instance, 90% of *A. baumannii* isolates from five medical centres in Vietnam were found to have gained multiple resistance to β-lactams, cephalosporins, aminoglycosides, and carbapenems [23]. Carbapenem resistance in *A. baumannii* is largely mediated via *bla*_OXA-23_ [[19], [20], [21], [22],^24^,^25^], whilst *bla*_NDM-1_ has sporadically been found [19,21]. The lack of vaccine against *A. baumannii* and the increasing resistance to carbapenems and last-line drugs underline the needs of closely monitoring the epidemiology and clinical effect of CRAB in Vietnam. Here, we conducted a prospective surveillance of *A. baumannii* infections at a tertiary hospital in Vietnam to characterise the clinical features of patients infected with CRAB and to dissect the molecular characteristics and pathogen population dynamics.

## 2 Material and methods

### 2.1 Study setting

The University Medical Center (UMC) is a large tertiary and teaching hospital in Ho Chi Minh City (HCMC), Vietnam. The hospital has ca. 1000 beds and provides healthcare services for more than 5000 outpatients per day, plus 55 000 inpatients and 30 000 surgeries per year. The UMC has 34 clinical wards and 10 subclinical wards, including an ICU with 30 beds. The majority of patients are from HCMC and its surrounding provinces.

### 2.2 Study design

Patients admitted to all clinical wards (ICU and non-ICU wards) at the UMC between October 2019 and October 2020 who had clinical diagnosis of bacterial infections and microbiological culture positive with *A. baumannii* from one of the following clinical specimens—blood, sputum/BAL, pus/wound swab, or urine—were eligible for the study. Patients’ guardians/caregivers were asked to provide written informed consent prior to enrolment. Upon patient enrolment, the following data were retrospectively collected from electronic health record: date of admission, age, sex, place of admission (home/hospital transfer), underlying conditions, patient location, prior antibiotic use, immunosuppressive therapy, and invasive procedures. Patients were followed until discharge or in-hospital death, and information regarding ICU stay, duration of hospital stay, discharge date, and outcome was recorded. In-hospital mortality was defined as deaths in hospital or patients discharged palliatively. The data were collected from all study participants using a case report form and subsequently deidentified and transferred to an electronic database.

For microbiological data, we collected information regarding sample type (blood, sputum/BAL, pus/wound swab, urine), dates of sample collection and positive culture, place of sample collection, clinical diagnosis, and antimicrobial susceptibility (AST) results. Hospital-acquired infection (HAI) was defined as positive culture with *A. baumannii* from a patient with clinically suspected infection at least 48 hours after hospital admission; community-acquired infection (CAI) was defined as positive culture with *A. baumannii* from a patient with clinically suspected infection within 48 hours of hospital admission and without a history of hospital transfer. Metadata associated with the study are included in Supplementary Table S1.

### 2.3 Microbiological culture

For blood culture, two to four bottles with 8–10 mL of blood per bottle were routinely obtained and inoculated into aerobic and anaerobic blood culture bottles, which were subsequently incubated at 35 ± 2°C in BACT/ALERT VIRTUO (bioMérieux, France) or BD BACTEC FX (Becton Dickenson, USA) automated analyser for up to five days. Subculture was performed on fresh sheep blood, MacConkey, and chocolate agars when the machine indicated a positive signal. Organisms were identified using a BD Phoenix M50 (Becton Dickenson, USA) or Vitek 2 Compact (bioMérieux, France) automated identification and AST testing systems. For sputum culture, sample quality was assessed using Bartlett's grading system [27], followed by plating onto selective media for bacterial isolation. For bronchoalveolar lavage aspirate (BAL) and urine culture, samples were quantitatively plated onto selective media, and bacterial identification and AST were performed for known pathogens from BAL with a colony count ≥10^4^ cfu/mL and uropathogens with a colony count ≥10^5^ cfu/mL. When multiple *A. baumannii* organisms were isolated from the same patient, only the first isolate was included for analyses.

### 2.4 Antimicrobial susceptibility testing

AST was performed by a BD Phoenix^TM^ M50 or Vitek 2 Compact automated system for routinely tested antibiotics. Broth microdilution method was used to measure the MICs against colistin following CLSI guidelines [28]. AST results were interpreted according to the CLSI 2019 guidelines [28]. CLSI breakpoints for colistin MIC ≥4 mg/L and ≤2 mg/L were considered resistant and susceptible, respectively. MDR was defined as resistance to at least one agent in three or more antimicrobial categories [29].

### 2.5 Whole genome sequencing

Genomic DNA from *A. baumannii* was extracted using the Wizard Genomic DNA Extraction Kit (Promega, USA), and 1 ng of genomic DNA from each sample was subjected to library preparation using a Nextera XT kit. WGS was performed using Illumina MiSeq (Illumina, USA) to generate 250 bp paired-end reads. Raw sequence data are available in the European Nucleotide Archive (Project number: PRJEB51471).

### 2.6 Gene content analysis and species identification

SRST2 v.0.2.0 [30] was used to identify acquired resistance genes, virulence genes, plasmid replicon types, and MLST using the following databases: ARG-ANNOT [31], Virulence Factor Database (VFDB: http://www.mgc.ac.cn/VFs/), customized plasmid replicon database for *Acinetobacter* [17], and *A. baumannii* MLST Pasteur scheme [32], respectively. Kaptive [33] was used to identify capsule polysaccharide (KL) and lipooligosaccharide outer core (OCL) types. All Illumina reads were de novo assembled using Unicycler v.0.4.8 [34] to generate contigs using the default settings. MASH [35] was used to generate Mash distances, a good approximation to ANI values, which were subsequently used to compute pairwise ANI-based distances between our *Acinetobacter* isolates and a published reference collection of *Acinetobacter* species [36]*.* The pairwise ANI with a 96% cutoff was used to confirm species.

### 2.7 SNP detection and phylogenetic analysis

Illumina trimmed reads were mapped against the reference genome, *Acinetobacter baumannii* ST2 WM99c (accession number: CP031743), using RedDog pipeline v.1.10b with default parameters [37]. Briefly, RedDog used Bowtie2 v.2.2.3 [38] to map all raw reads to the reference sequence, and high-quality SNPs with Phred quality score ≥30 were extracted using SAMtools v1.3.1 [37]. SNPs were filtered to remove those with fewer than five supporting reads or with >2.5 times the mean read depth (representing putative repeated sequences), or with ambiguous base calls. A pseudo-genome alignment was inferred using the above reference sequence with the snpTable2GenomeAlignment.py script from RedDog. Genomic sequences were removed from further analyses if there was evidence suggestive of contamination, i.e., <50% of mapped reads or the total assembly length being >4.5 Mb. The pseudo-whole-genome alignment was subjected to Gubbins v.1.4.5 [39] for recombination removal, and SNP-sites [40] were used to extract SNPs from the recombination-free multi-FASTA alignment, resulting in an alignment of 153 nonrecombinant SNPs between ST2 isolates. IQ-tree v.1.3.1 [41] was used to run model testing and infer a maximum likelihood (ML) phylogenetic tree using the best-fit nucleotide substitution model, K2P+ASC. The ML tree was rooted using the WM99 strain as an outgroup. Support for the ML tree was assessed via 1000 pseudo-replicates. SNPPar [42] was used to identify SNPs occurring on ST2 phylogenetic branches. To provide further contextualization of ST2 circulation, we combined our genomic data with previously published ST2 genomes from other hospitals in Vietnam (*n* = 64) (Supplementary Table S2) and reconstructed a secondary phylogenetic tree using the same method as above.

SNP detection was performed for ST571 and ST16 isolates using the same approach for ST2 isolates, yielding an alignment of nonrecombinant SNPs of 209 sites for ST571 and 103 sites for ST16 isolates. The ML tree for ST571 isolates was also reconstructed using IQ-tree v.1.3.1 and rooted with the WM99c strain as an outgroup.

### 2.8 Statistical analyses

Statistical analyses were conducted using R (v.4.1.2). Pearson's χ^2^ or Fisher's exact test was used for categorical variables, and Mann-Whitney test for continuous data. Univariate analyses were first performed to identify significant variables. To avoid overfitting, only variables that were significant in univariate analysis (*P* < 0.05) were included in the multivariate logistic regression model. The most significant variables were then selected using a stepwise approach until the best fitted model was obtained. The preferred multivariate model was the one with the minimum Akaike information criterion value.

## 3 Results

### 3.1 Demographic and clinical characteristics of A. baumannii (AB) infections

A total of 84 patients were enrolled in the study. Baseline demographics and clinical characteristics of AB cases are shown in Table 1. The median age of the patients was 73.5 years (IQR: 64–86 years). Nearly 55% of the study population was male. The majority of patients (81%) had at least one underlying condition. Half of the patients (50%) acquired *A. baumannii* infections while staying in the ICU, with a median duration from ICU admission to the development of the infection of 9.5 days (IQR: 4–17.5 days). Among non-ICU patients, the median time between hospital admission and AB infection was 8 days (IQR: 2–16.5 days). Previous antibiotic use was common, with 88.1% of patients having received at least one antibiotic; the most frequently used drugs were carbapenems (70.2%), glycopeptides (44.1%), fluoroquinolones (41.7%), third-generation cephalosporins (29.8%), and linezolid (27.4%). Previous exposure to immunosuppressive therapy was found in 61.9% of patients. The usage of invasive procedure was 60.7% for mechanical ventilation, 29.8% for central venous catheterization, and 32.1% for urinary catheterization. The most common site of AB isolation was the lower respiratory tract (64.3%), followed by pus/wound swabs (20.2%), urine (10.7%), and blood (4.8%). Carbapenems (76.2%), glycopeptides (44.0%), colistin (41.7%), third-generation cephalosporins (38.1%), fluoroquinolones (32.1%), and linezolid (32.1%) were the most common drugs used following the identification of AB infections.

**Table 1:** Demographic and clinical characteristics of *A. baumannii* infections stratified by in-hospital mortality

	Nonsurvivors (N = 47)	Survivors (N = 37)	Total (N = 84)	*P* value
Age				0.018
Median years, (IQR)	83 (68.5–86)	67 (53.0–82.0)	73.5 [64–86]	
Sex, male	24 (51.1%)	22 (59.5%)	46 (54.8%)	0.443
ICU admission prior to the event	34 (72.3%)	8 (21.6%)	42 (50.0%)	< 0.001
Length of stay				0.878
Mean days ± SD	37.4 ± 35.1	28.7 ± 16.2	33.6 ± 28.6	
Median days (IQR)	25 (14.5–46.5)	26 (17.0–36.0)	25.5 (15.8–37.8)	
Underlying conditions	37 (78.7%)	31 (83.8%)	68 (81.0%)	0.728
Source of AB isolation				0.009
Lower respiratory tract	37 (8.7%)	17 (5.9%)	54 (64.3%)	0.002
Blood	2 (4.3%)	2 (5.4%)	4 (4.8%)	0.999
Pus/wound swab	6 (12.8%)	11 (29.7%)	17 (20.2%)	0.055
Urine	2 (4.3%)	7 (18.9%)	9 (10.7%)	0.039
Immunosuppressive therapy	35.00 (74.5%)	17 (45.9%)	52.00 (61.90%)	0.008
Invasive procedures				
Mechanical ventilation	37 (78.7%)	14 (37.8%)	51 (60.7%)	<0.001
Central venous catheterization	19 (40.4%)	6 (16.2%)	25.0 (29.8%)	0.017
Urinary catheterization	14.0 (29.8%)	13.0 (35.1%)	27.0 (32.1%)	0.602
Others	25.0 (53.2%)	15.0 (40.5%	40.0 (47.6%)	0.249
Antibiotic use before AB infection				
Third generation cephalosporins	17 (36.2%)	8 (21.6%)	25 (29.8%)	0.148
Carbapenems	35 (74.5%)	24 (64.9%)	59 (70.2%)	0.339
Glycopeptides	24 (51.1%)	13 (35.1%)	37 (44.0%)	0.144
Fluoroquinolones	19 (40.4%)	16 (43.2%)	35 (41.7%)	0.795
Colistin	9 (19.1%)	3 (8.1%)	12 (14.3%)	0.213
Linezolid	18 (38.3%)	5 (13.5%)	23 (27.4%)	0.014
Aminoglycosides	9 (19.1%)	1 (2.7%)	10 (11.9%)	0.037
Macrolides	6 (12.8%)	1 (2.7%)	7 (8.3%)	0.128
Penicillins with beta-lactamase inhibitors	10 (21.3%)	8 (21.6%)	18 (21.4%)	0.969
Antibiotic use after AB infection				
Third-generation cephalosporins	6.0 (12.8%)	6.0 (16.2%)	12.0 (14.3%)	0.654
Carbapenems	37 (78.7%)	27 (73.0%)	64 (76.2%)	0.539
Glycopeptides	22 (46.8%)	15 (40.5%)	37 (44.0%)	0.566
Fluoroquinolones	17 (36.2%)	10 (27.0%)	27 (32.1%)	0.373
Linezolid	17 (36.2%)	10 (27.0%)	27 (32.1%)	0.373
Colistin	26 (55.3%)	9 (24.3%)	35 (41.7%)	0.004
Aminoglycosides	8 (17.0%)	2 (5.4%)	10 (11.9%)	0.174
Macrolides	6 (12.8%)	3 (8.1%)	9 (10.7%)	0.725
Penicillins with beta-lactamase inhibitors	15.0 (31.9%)	14.0 (37.8%)	29.0 (34.5%)	0.647
Fosfomycin	1 (2.1%)	2 (5.4%)	3 (3.6%)	0.58
CRAB	45 (95.7%)	31 (83.8%)	76 (90.5%)	0.130
Sequence types				0.034
ST2	32 (68.1%)	16 (45.7%)	48 (58.5%)	
ST571	8 (17.0%)	5 (14.3%)	13 (15.9%)	
Other STs	7 (14.9%)	14 (40%)	21 (25.6%)	
Missing	0	2	2	

Three cases were identified as community-acquired infections, while the remaining 81 cases were hospital-acquired infections. The mean length of hospital stay was 33.6 ± 28.6 days. The overall in-hospital mortality rate was 56% (47/84); however, the mortality rate increased to 80.9% (34/42) among ICU-acquired cases. Compared with the survivor group, patients in the nonsurvivor group were strongly associated with advanced age (*P* = 0.018), ICU admission (*P* < 0.001), lower respiratory tract as the origin of AB (*P* = 0.002), exposures to immunosuppressive drugs (*P* = 0.008), mechanical ventilation (*P* < 0.001), central venous catheterization (*P* = 0.017), and previous uses of aminoglycosides (*P* = 0.037), linezolid (*P* = 0.014), and AB treatment with colistin (*P* = 0.004) (Table 1).

### 3.2 Risk factors for in-hospital mortality in patients with A. baumannii infections

We performed univariate and multivariate logistic regression analyses to identify risk factors associated with in-hospital mortality. Univariate analyses showed that advanced age (*P* = 0.025), ICU admission (*P* < 0.001), lower respiratory tract as the source of AB (*P* = 0.002), immunosuppressive therapy (*P* = 0.009), mechanical ventilation (*P* < 0.001), central venous catheterization (*P* = 0.019), and previous uses of linezolid (*P* = 0.015), aminoglycosides (*P* = 0.047), and colistin-based treatment (*P* = 0.005) were significantly associated with in-hospital mortality. Multivariate analysis revealed that ICU admission (*P* = 0.001) was the only independent predictor of in-hospital deaths in patients with *A. baumannii* infections (Table 2).

**Table 2:** Univariate and multivariate logistic regression of risk factors associated with in-hospital mortality in AB patients

Characteristics	Univariate			Multivariable		
	OR	95% CI	*P* value	OR	95% CI	*P* value
Age	1.03	1.01, 1.06	0.025	1.03	1.00, 1.06	0.105
ICU admission	9.99	3.79, 28.7	<0.001	6.03	2.10, 18.4	0.001
Sources of AB isolation						
Lower respiratory tract	4.35	1.72, 11.7	0.002	2.75	0.89, 8.77	0.08
Urine	0.19	0.03, 0.85	0.047			
Immunosuppressive therapy	3.43	1.39, 8.84	0.009			
Invasive procedures						
Mechanical ventilation	6.08	2.38, 16.6	<0.001			
Central venous catheterization	3.51	1.28, 10.8	0.019			
Antibiotic use before AB infection						
Linezolid	3.97	1.39, 13.3	0.015			
Aminoglycosides	8.53	1.49, 161	0.047	8.41	0.97, 203	0.096
Antibiotic use after AB infection						
Colistin	3.85	1.54, 10.3	0.005			

CI, confidence interval; OR, odds ratio.

### 3.3 Antimicrobial susceptibility of A. baumannii isolates

Fifty percent of the isolates (42/84) were identified from patients residing in ICU, while the remaining isolates were from 10 different clinical wards. About 90.5% of isolates were resistant to carbapenems. The resistance rates to fluoroquinolone, third-generation cephalosporins, aminoglycosides, beta-lactams/beta-lactam inhibitor, and trimethoprim/sulfamethoxazole were 90.5%, 92.9%, 86.9%, 88.1%, and 82.7%, respectively. Overall, 92% of isolates were multidrug-resistant. Five isolates (6%) were resistant to colistin, four of which were also resistant to carbapenems. The colistin MIC50 and MIC90 values were 0.5 mg/L and 1 mg/L, respectively. Infections with CRAB isolates were not associated with the nonsurvivor group (*P* = 0.13).

### 3.4 Genomic analysis of A. baumannii isolates

WGS data from 82 microbiologically defined *A. baumannii* isolates were available for analyses. Our data showed that 76 isolates were confirmed as *A. baumannii*; other species included *A. pittii* (4), *A. nosocomialis* (1), and *A. seifertii* (1). Fifteen STs were identified, of which the dominant STs were ST2 (58.5%), ST571 (15.9%), and ST16 (4.9%); each of the 12 remaining STs were represented by one to three isolates. ST2 isolates were hospital-wide distributed, encompassing ICU (62.5%) and other eight clinical wards. About 50% of the ST571 isolates were detected in ICU, while all ST16 isolates were from non-ICU wards. For the three *A. baumannii* isolates from CAIs, all of them were resistant to carbapenem and belonged to ST2, ST132, and ST571.

All *A. baumannii* isolates harboured the chromosomal *bla*_OXA-51-like_ gene, 88.2% (67/76) of which also carried the carbapenem resistance gene *bla*_OXA-23_. *Bla*_NDM-1_ was found in eight isolates, including five *A. baumannii* (ST16: four isolates; ST32: one isolate) and three *A. pittii* isolates (ST207: two isolates; ST220: one isolate). Three *bla*_NDM-1_-carrying isolates also possessed *bla*_OXA-58_. Notably, the three predominant STs carried multiple but distinct AMR gene profiles, for example: *bla_OXA-23_-mphE-msrE-aph3′Ia*(+/-)*-armA-strA-strB-sulIII*(+/-)*-bla_TEM_-tetB* for ST2; *bla_OXA-23_-mphE-msrE-aadA-armA-sulI-bla_TEM_* for ST571 and *aac3-IId*(+/-)*-aphA6-mphE*(+/-)*-msrE*(+/-)*-bla_NDM-1_-bla_OXA-58_*(+/-)*-tet39*(+/-) for ST16*.* The distribution of key virulence factors was similar between the three STs; the exceptions were the absence of abaI-abaR (quorum sensing) in ST571 isolates, and the lack of bap (biofilm-associated protein), hemO (heme utilization) and galU-pgi (polysaccharide synthesis) in ST16 isolates. No plasmid replicon was found in any isolates.

The five colistin-resistant isolates belonged to *A. baumannii*-ST2 (1), *A. baumannii*-ST571 (2), *A. pittii*-ST207 (1), and *A. seifertii*-STNF (1). Colistin resistance was mediated by an insertion of ISAba1 upstream of the *eptA* gene (position +51) encoding phospho-ethanolamine transferase in *A. baumannii*-ST2; nonsynonymous mutation P170L in the *pmrB* gene in *A. baumannii*-ST571, inactivation of lipid A biosynthesis genes *lpsB, lpxC, lpxD, lpxL, lpxM*, and/or *lpxA* and/or *lpxB* in *A. pittii*-ST207 and *A. seifertii*-STNF.

### 3.5 Phylogenetic analysis of A. baumannii ST2 and ST571 isolates

**Fig. 1:**
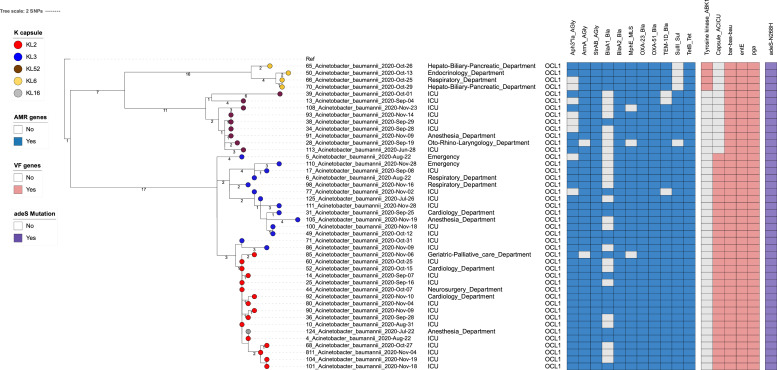
Phylogenetic structure of *Acinetobacter baumannii* ST2 isolates from University Medical Center, Vietnam. Maximum likelihood (ML) phylogeny of *A. baumannii* ST2 from University Medical Center in Ho Chi Minh City, Vietnam. The ML tree was rooted using *A. baumannii* ST2 strain WM99c as an outgroup. The terminal nodes are coloured according to capsular polysaccharide type (KL types) of ST2 isolates. The scale bar shows the number of SNPs. The heat map shows the presence (blue and red colour) or absence (grey colour) of acquired antimicrobial resistance genes and virulence factors.

**Fig. 2:**
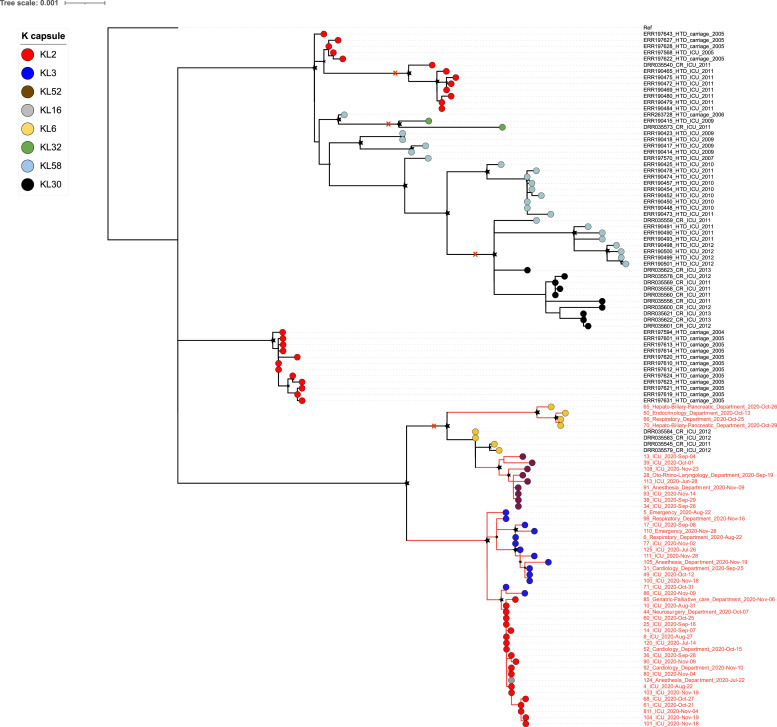
Phylogenetic structure of *Acinetobacter baumannii* ST2 isolates from this study together with previously published ST2 genomes from Vietnam. Maximum likelihood (ML) phylogeny of *A. baumannii* ST2 from this study together with previously published ST2 genomes from other hospitals in Vietnam. The ML tree is rooted using *A. baumannii* ST2 strain WM99c as an outgroup. The terminal nodes are coloured according to capsular polysaccharide type (KL types) of ST2 isolates. The scale bar shows the number of substitutions per site. Black stars indicate bootstrap support values ≥80% on internal nodes, with larger stars indicating higher bootstrap values. Red cross indicates clusters containing isolates from different hospitals.

We reconstructed the phylogeny of *A. baumannii* ST2 isolates and mapped it against patients’ treatment wards to investigate the circulation of these organisms. The ST2 isolates were separated into four different phylogenetic clusters, largely corresponding to four KL types: KL2, KL3, KL6, and KL52. The KL6 cluster was solely found in non-ICU wards, whereas the other three clusters were found in both ICU and non-ICU wards. Furthermore, the ICU and non-ICU isolates clustered together in monophyletic clusters with a maximum genetic distance of ≤5 SNPs, providing strong evidence for between-ward transmissions, given that the substitution rate of *A. baumannii* GC2 was estimated to be ∼10 SNPs/year [19], and a cutoff ≤10 SNPs was used to identify *A. baumannii* transmission in an ICU [43]. Our data revealed the co-circulation of multiple ST2 clusters with distinct KL types in ICU, as well as intermittent spread of ST2 organisms between ICU and non-ICU wards (Fig. 1, Supplementary Fig. S1). Notably, we identified a capsular switch between KL3 and KL2, mediated by a recombination event in the K locus. According to our rooted phylogenetic tree, the KL3 cluster was more basal to the most recent common ancestor of KL3 and KL2 isolates; additionally, the mean pairwise SNP distance between KL2 isolates was significantly lower than that of KL3 isolates (1.9 SNPs vs. 7.8 SNPs) (*P* < 0.001). These data indicated the capsule switching was probably from KL3 to KL2. To provide further contextualization of ST2 transmission, we compared our data with previously published ST2 genomes from other hospitals in southern Vietnam. We found several occasions when ST2 isolates from different hospitals clustered together, demonstrating between-hospital transmissions of these organisms. Additionally, capsule switching appeared to occur rather frequently during the circulation of this clone (Fig. 2).

For ST571 isolates, most isolates (10/13) belonged to a KL10 phylogenetic cluster, comprising both ICU and non-ICU isolates. Within this cluster, there were two isolates from the ICU and respiratory ward differing by only one SNP, suggesting occasional transmission of ST571 between the two treatment wards (Supplementary Fig. S2).

## 4 Discussion

Here, we identified several potential risk factors associated with in-hospital mortality in patients infected with *A. baumannii*, including those that had been previously described in Vietnam and other countries, such as advanced age [5], ICU stay [44], exposure to immunosuppressive therapy [45], use of mechanical ventilation and central venous catheterization [6,44,45], and AB lower respiratory tract infections [4,6,44]. Furthermore, the prior use of antibiotics in our study population was very common, among which the use of linezolid or aminoglycosides was significantly higher in nonsurvivors. Despite this interesting observation, the results should be interpreted with caution given the small number of patients. Furthermore, aminoglycosides and linezolid were not included in empirical antibiotic regimens for *A. baumannii* in our setting, and their usage may have been associated with severe patients with multiple infections or patients who did not respond to existing/previous treatment therapy and thus were prone to develop poor outcomes. We did not collect the information about other bacterial infections before the acquisition of *A. baumannii*, which may have resulted in the usage of aminoglycosides and linezolid.

The global spread of CRAB has prompted the increased use of colistin either alone or in combination with other drugs (i.e., meropenem, sulbactam, fosfomycin) for treatment [46]. Our study suggests that colistin-based treatment is a predictor of deaths in hospital, which is similar to the finding from a previous study [47]. Although the attributable causes of mortality in colistin-treated patients were difficult to ascertain, the use of colistin should be judicious and the dosing regimens must be carefully selected and monitored given its association with nephrotoxicity and neurotoxicity. Alternatively, non-colistin antimicrobial therapies effective against CRAB, such as tigecycline [48] and sulbactam [46], may also be considered. Furthermore, new antimicrobial agents with activities against CRAB that have been either FDA-approved (cefiderocol) or under clinical development (new beta-lactam/beta-lactam inhibitors combinations: durlobactam + sulbactam, zidebactam + cefepime) may be essential to treat CRAB [49]. Having access to these new antibacterial drugs and evaluating their clinical performance will be essential to provide new treatment of CRAB infections in Vietnam.

We found an extremely high prevalence of carbapenem resistance in *A. baumannii*, mainly mediated by the acquisitions of *bla_OXA-23_*; however, *bla_NDM-1_* was also detected. The presence of distinct combinations of beta-lactamase and carbapenemase genes (*bla_OXA-23_*/*bla_NDM-1_*) in CRAB may result in decreased susceptibility to the new beta-lactam/beta-lactam inhibitors combinations; thus, in vitro testing of these new drugs against distinct CRAB genotypes is warranted to predict in vivo effectiveness. In addition to carbapenem resistance, the MDR prevalence was extremely high; our finding concurred with published data from Vietnam demonstrating an alarming rate of carbapenem plus MDR in *A. baumannii* [19,21,22,24,25]. We also found a small proportion of colistin-resistant *Acinetobacter* isolates (6%). Our work highlights the imminent threat of untreatable AB infections in Vietnam and calls for further research to limit the spread of XDR organisms and to evaluate in vitro activity of new antibiotics against CRAB.

We found strong evidence of between-ward and between-hospital transmissions of CRAB ST2, probably mediated by sharing of medical equipment and/or patient transfers between wards and hospitals. Our study corroborates the findings from previous studies that have shown a dominance and potential country-wide expansion of ST2 in Vietnam [19,50] and Southeast Asia [17]. Regular capsular switch (including the KL3 to KL2 event) within the ST2 population was also evident, which potentially results in immune escape or enhanced colonization and infections in susceptible hosts. Capsular polysaccharide plays an important role in virulence, AMR, and environmental persistence of *A. baumannii* [51,52], and a previous study has also found that replacement of a capsular locus is a key determinant of population changes [19]. We hypothesize that capsular diversification is a key evolutionary factor shaping the population structure and dynamics of CRAB ST2.

Our study has some limitations. The study was subjected to several disruptions due to the COVID-19 pandemic; hence, the number of study participants was lower than expected. Our findings were restricted to a single healthcare centre encompassing a limited number of patients, and results may not be applicable to other hospital settings in Vietnam. Most deaths occurred in ICU, where patients had prolonged stays before developing AB infections and were exposed to various risk factors; thus, the contribution of CRAB infections to the development of mortality was difficult to determine.

In conclusion, we found *A. baumannii* infections were most prevalent in ICU, but also were found in non-ICU wards. Most infections were hospital-acquired; various potential risk factors associated with in-hospital mortality were characterised. The prevalence of carbapenem resistance and MDR in *A. baumannii* was extremely high, together with the emergence of colistin resistance. ST2, ST571, and ST16 were the top three dominant genotypes identified, carrying a diverse array of AMR genes conferring carbapenem resistance plus MDR phenotype. We found evidence of within- and between-hospital transmissions and clonal diversification via capsular switching of CRAB ST2. Strengthening hospital infection control measures and routine surveillance is key to limit the spread of these organisms and to detect of the emergence of novel pan-drug-resistant variants in a timely fashion.

## Funding

Duy Thanh Pham is funded by a Wellcome Trust International Training Fellowship (222983/Z/21/Z). Duong Thi Hong Diep is funded by the University of Medicine and Pharmacy in Ho Chi Minh City, Vietnam (number 5676/QĐ-ĐHYD). The funders had no role in the design and conduct of the study; collection, management, analysis, and interpretation of the data; preparation, review, or approval of the manuscript; or decision to submit the manuscript for publication.

## Ethical approval

The study received approval from the Ethics Committee of UMP in HCMC (approval number: 283/HĐĐĐ-ĐHYD).

## Competing interests

All authors declare that there are no conflicts of interests.

## Appendix Supplementary materials

Supplementary Figure 1. Spatio-temporal presentation of genetically clusters within ST2 isolates Kernel density estimation of the spatial temporal distribution of KL clusters within *A. baumannii* ST2 phylogeny. The height of the curve is chosen so that the area under the curve is one. Gaussian kernel and a bandwidth value of 15 are utilized to estimate the density. Each dot represents an isolate within each of the KL clusters and is colored according to patient's location (ICU versus non-ICU wards). Image, application 1

Supplementary Figure 2. Phylogenetic structure of *A. baumannii* ST571 isolates The ML tree is rooted using *Acinetobacter baumannii* ST2 strain WM99c as an outgroup. The terminal nodes are colored according to capsular polysaccharide type (KL types) of ST571 isolates. The scale bar shows the number of SNPs. The heat map shows the presence (blue and red color) or absence (grey color) of acquired antimicrobial resistance genes and virulence factors. Image, application 2

Supplementary Table 1: Metadata associated with A. baumannii isolates in our study Image, application 3

Supplementary Table 2: Metadata associated with previously published *A. baumannii* ST2 isolates from Hospital for Tropical Diseases (HTD) and Cho Ray hospital (CR) in HCMC, Vietnam Image, application 4

Supplementary Table 3: Quality assessment of whole genome sequencing data Image, application 5

Image, application 6
