# Supplementary figures and images for: The clinical features and genomic epidemiology of carbapenem-resistant Acinetobacter baumannii infections at a tertiary hospital in Vietnam
Source: J Glob Antimicrob Resist. 2023 Jun;33:267–75. doi: 10.1016/j.jgar.2023.04.007 (PMC10275762; doi:10.1016/j.jgar.2023.04.007)

KL

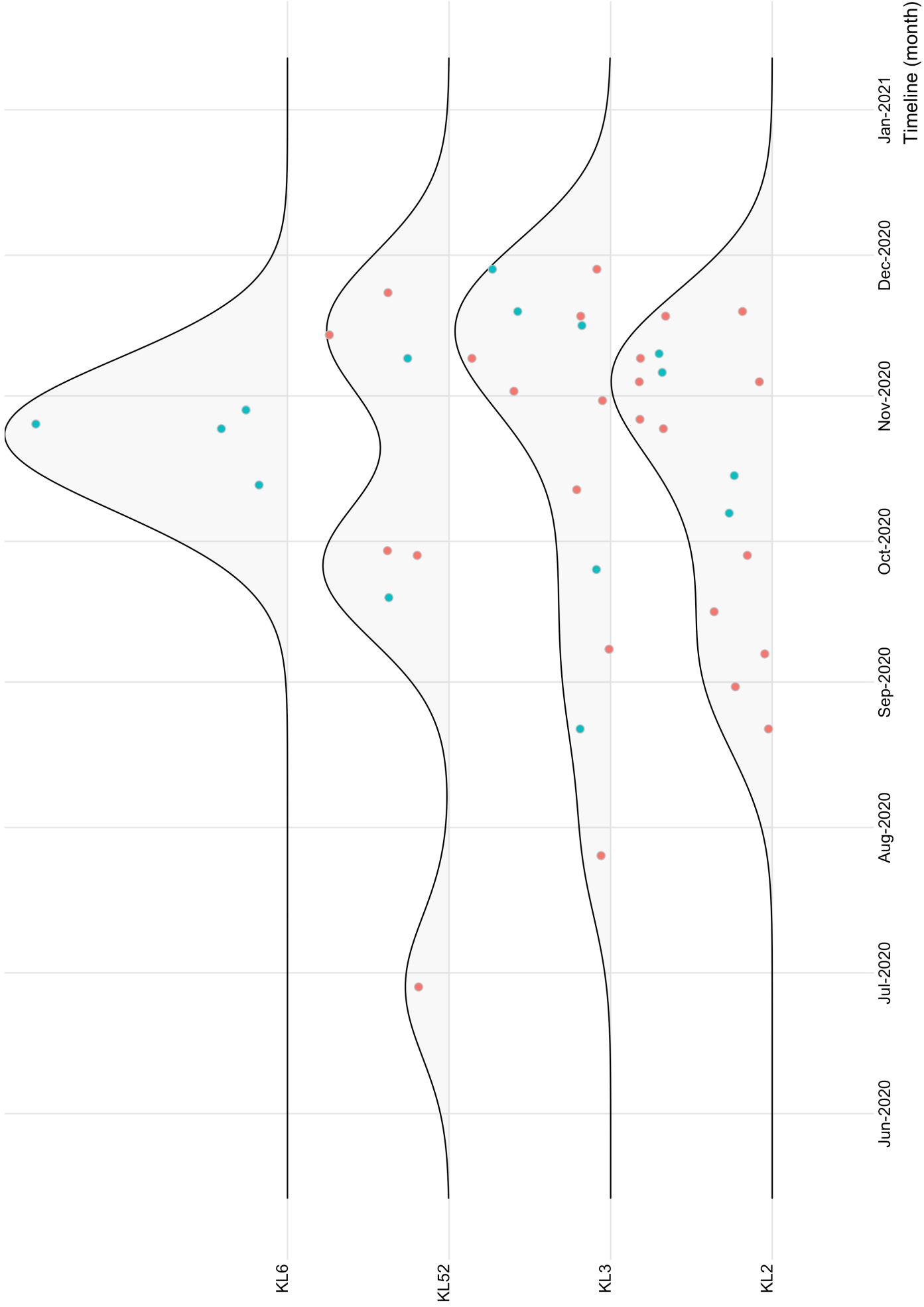

Supplement: Supplementary file 1 — Supplementary Figure 1. Spatio-temporal presentation of genetically clusters within ST2 isolates Kernel density estimation of the spatial temporal distribution of KL clusters within A. baumannii ST2 phylogeny. The height of the curve is chosen so that the area under the curve is one. Gaussian kernel and a bandwidth value of 15 are utilized to estimate the density. Each dot represents an isolate within each of the KL clusters and is colored according to patient's location (ICU versus non-ICU wards). [file mmc1.pdf]

Tree scale:4snps

## K capsule

- KL58
- KL10
- KL49

## AMR genes

☐ No  
☒ Yes

## VF genes

☐ No  
☒ Yes

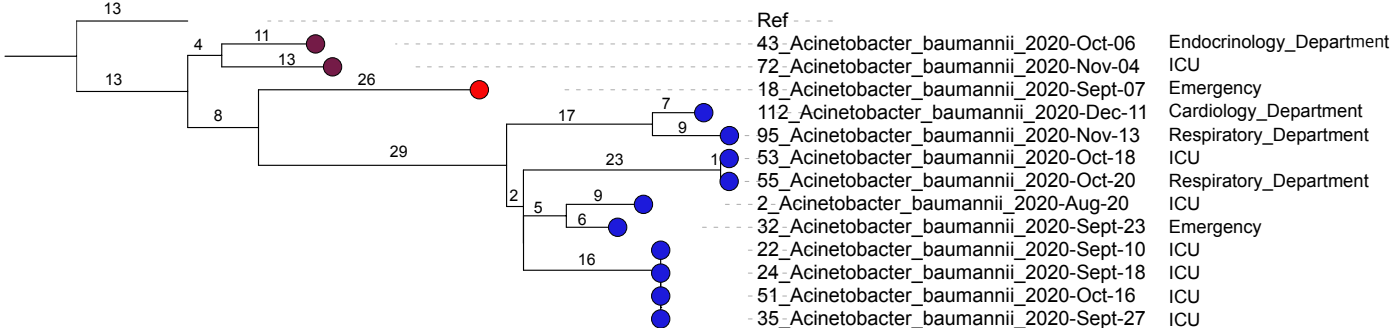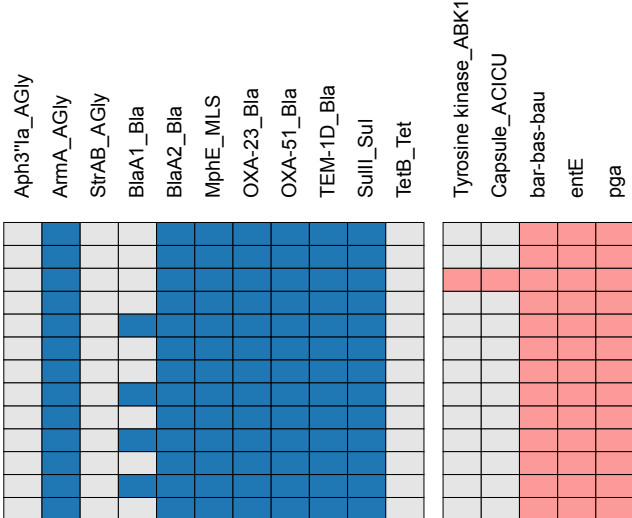

Supplement: Supplementary file 2 — Supplementary Figure 2. Phylogenetic structure of A. baumannii ST571 isolates The ML tree is rooted using Acinetobacter baumannii ST2 strain WM99c as an outgroup. The terminal nodes are colored according to capsular polysaccharide type (KL types) of ST571 isolates. The scale bar shows the number of SNPs. The heat map shows the presence (blue and red color) or absence (grey color) of acquired antimicrobial resistance genes and virulence factors. [file mmc2.pdf]
