# Supplementary material for: The clinical features and genomic epidemiology of carbapenem-resistant Acinetobacter baumannii infections at a tertiary hospital in Vietnam
Source: J Glob Antimicrob Resist. 2023 Jun;33:267–75. doi: 10.1016/j.jgar.2023.04.007 (PMC10275762; doi:10.1016/j.jgar.2023.04.007)
Supplement: Supplementary file 6 [file mmc6.docx]

**Supplementary data**

Tables S1, S2 are available as Supplementary data online.

**Supplementary Figure 1. Spatio-temporal presentation of genetically clusters within ST2 isolates**

Kernel density estimation of the spatial temporal distribution of KL clusters within *A. baumannii* ST2 phylogeny. The height of the curve is chosen so that the area under the curve is one. Gaussian kernel and a bandwidth value of 15 are utilized to estimate the density. Each dot represents an isolate within each of the KL clusters and is colored according to patient’s location (ICU versus non-ICU wards).

**Supplementary Figure 2. Phylogenetic structure of *A. baumannii* ST571 isolates**

The ML tree is rooted using *Acinetobacter baumannii* ST2 strain WM99c as an outgroup. The terminal nodes are colored according to capsular polysaccharide type (KL types) of ST571 isolates. The scale bar shows the number of SNPs. The heat map shows the presence (blue and red color) or absence (grey color) of acquired antimicrobial resistance genes and virulence factors.

**Supplementary Table 1: Metadata associated with *A. baumannii* isolates in our study**

**Supplementary Table 2: Metadata associated with previously published *A. baumannii* ST2 isolates from Hospital for Tropical Diseases (HTD) and Cho Ray hospital (CR) in HCMC, Vietnam**

**Supplementary Table 3: Quality assessment of whole genome sequencing data**
